# Supplementary material for: Impact of Social Needs and Identity Experiences on the Burden of Illness in Patients with Multiple Myeloma: A Mixed-Methods Study
Source: Healthcare (Basel). 2024 Aug 20;12(16):1660. doi: 10.3390/healthcare12161660 (PMC11353550; doi:10.3390/healthcare12161660)
Supplement: Supplementary file 1 [file healthcare-12-01660-s001.zip › healthcare-3097912-supplementary.pdf]

Supplemental Materials

Supplemental Material S1. Social determinants of health survey questionnaire

Let's begin with some basic questions about you.

1. What is your first name? **[OPEN FIELD]**

2. What is your last name? **[OPEN FIELD]**

3. What is your preferred name? **[OPEN FIELD]**

4. What is your education level?
   () Less than High School
   () High School or GED
   () Some college
   () Technical or trade school
   () Associate's Degree
   () Bachelor's Degree
   () Graduate Degree (e.g., Master's Degree, PhD, MD)

5. Of the following descriptions, which best fits the community where you currently live?
   () Urban: Urban areas consist of both living and working areas with a high population or are zip codes in large cities
   () Suburban: Suburban areas are areas that are mainly residential area with a larger population than rural areas
   () Rural: Rural areas are areas that are open and spread out with a small population

6. What is your current employment status? Please select **ALL** that apply. **[ALLOW MULTIPLE RESPONSES]**
   [] Employed full-time
   [] Employed part-time
   [] Unemployed
   [] Stay-at-home parent
   [] Self-employed
   [] Student
   [] Retired
   [] Other (Please specify) **[OPEN FIELD]**

7. Gender. Please select **ALL** that apply. (Optional) **[ALLOW MULTIPLE RESPONSES]**
   [] Male
   [] Female
   [] Genderqueer/Nonbinary
   [] Other (Please specify) **[OPEN FIELD]**

8. Do you identify as transgender, nonbinary, or gender nonconforming? (Optional)
   () No
   () Yes (Please specify) **[OPEN FIELD]**

9. What pronouns do you prefer? (Optional)

() She/her/hers
() He/him/his
() They/them/theirs
() Ze/zir/zirs
() Other (Please specify) **[OPEN FIELD]**

10. What is your sexual orientation? (Optional)
    () Heterosexual/straight
    () Bisexual
    () Pansexual
    () Gay/lesbian
    () Other (Please specify) **[OPEN FIELD]**

11. Which category describes your race? Please select **ALL** that apply. (Optional) **[ALLOW MULTIPLE RESPONSES]**
    [] Asian or Asian-American (e.g., Chinese, Filipino, Asian Indian, Vietnamese, Korean, Japanese)
    [] Black or African American (e.g., African American, Jamaican, Haitian, Nigerian, Ethiopian, Somalian)
    [] Hispanic, Latino, or Spanish origin (e.g., Mexican or Mexican American, Puerto Rican, Cuban, Dominican, Salvadoran, Colombian)
    [] Native Hawaiian or Other Pacific Islander (e.g., Native Hawaiian, Samoan, Guamanian or Chamorro, Tongan, Fijian, Marshallese)
    [] Native peoples/Indigenous peoples/American Indian and/or Alaska Native (e.g., Navajo Nation, Blackfeet Tribe, Muscogee (Creek) Nation, Mayan, Doyon)
    [] White, (e.g., German, Irish, English, Italian, Lebanese, Egyptian, and so on)
    [] Other (Please specify) **[OPEN FIELD]**

12. Are you of Hispanic, Latino, or Spanish origin?
    () No, not of Hispanic, Latino or Spanish origin
    () Yes, Mexican, Mexican American, Chicano
    () Yes, Puerto Rican
    () Yes, Cuban
    () Yes, another Hispanic, Latino or Spanish origin (e.g., Argentinean, Columbian, Dominican, Nicaraguan, Salvadorian, Spaniard) (Please specify) [OPEN FIELD]

13. When you consider your race and/or ethnicity, how do you prefer to be identified? (e.g., Does Latinx resonate with how you identify yourself?) **[OPEN FIELD]**

In addition to your primary PERC condition, we would like to understand other conditions that you may have.

14. Have you been diagnosed with any of the following medical conditions? Please select **ALL** that apply. **[ALLOW MULTIPLE RESPONSES]**
    [] Autoimmune disease (e.g., but not limited to, Rheumatoid Arthritis, Lupus, Celiac Disease, Multiple Sclerosis, Ankylosing Spondylitis)
    [] Cancer

[] Cardiovascular disease (e.g., but not limited to, Coronary Artery Disease, Heart Attack, Arrythmia, High Blood Pressure)
[] Diabetes
[] Respiratory disease (e.g., but not limited to, COPD, Chronic Bronchitis, Emphysema, Cystic Fibrosis)
[] Mental health (e.g., but not limited to, depression or anxiety)
[] Overweight/obesity
[] Other (Please specify) **[OPEN FIELD]**

15. Are you currently enrolled in any of the following types of health insurance or health coverage plans? Please select **ALL** that apply. **[ALLOW MULTIPLE RESPONSES]**
[] Insurance through a current or former employer
[] Insurance through a spouse or partner's employer
[] Insurance purchased directly from an insurance company through a marketplace/exchange (e.g., self-employed)
[] Medicare, for people 65 and older or people with certain disabilities **[SKIP TO Q20]**
[] Medicaid or any kind of government-assistance plan for those with low incomes or a disability **[SKIP TO Q20]**
[] TRICARE or other military health care
[] VA (enrolled for health care through the Veteran's Administration)
[] Uninsured
[] Other (Please specify) **[OPEN FIELD]**

16. **[SKIP IF MEDICARE OR MEDICAID SELECTED IN Q15]** Have you heard of co-pay assistance and/or copay cards? **Definition:** Manufacturer copay cards are savings programs offered by pharmaceutical companies. They are also referred to as copay savings programs, copay coupons, or copay cards. They help insured patients afford expensive prescription drugs by reducing their out-of-pocket costs.
() Yes
() No

17. **[SKIP IF MEDICARE OR MEDICAID SELECTED IN Q15]** In the past, have you received co-pay assistance and/or copay cards?
() Yes
() No

18. **[SKIP IF MEDICARE OR MEDICAID SELECTED IN Q15]** In the calendar year 2022, how much in total did you receive in co-pay assistance and/or copay cards?
() $100 or less
() $101-200
() $201-300
() $301-400
() $401 or more
() I'm not sure

19. **[SKIP IF MEDICARE OR MEDICAID SELECTED IN Q15]** In the calendar year 2022, how many months were you enrolled in co-pay assistance?
() 1-3 months

() 4-6 months
() More than 6 months
() I'm not sure

20. How often do you need assistance understanding forms or other information given to you by a healthcare provider?
    () Often
    () Sometimes
    () Rarely
    () Never

21. How easy or difficult is it for you to navigate the healthcare system to obtain medical care or services?
    () Very difficult
    () Difficult
    () Neither difficult nor easy
    () Easy
    () Very easy

22. Have you ever filed for personal bankruptcy due in part to the impacts of your health conditions?
    () Yes
    () No

23. Are you currently on Supplemental Security Income (SSI) Disability?
    () Yes
    () No

24. What is your current living situation? (Optional)
    () I have a stable place to live
    () I have a place to live today, but **I am worried** about losing it in the future
    () I do not have a stable place to live (I am temporarily staying with others, in a hotel, in a shelter, living outside on the street, on a beach, in a car, abandoned building, bus or train station, or in a park)

25. Thinking about the place you live, do you have issues with any of the following? Please select **ALL** that apply. **[ALLOW MULTIPLE RESPONSES]**
    [] Pests such as bugs, ants, or mice
    [] Mold
    [] Lead paint or pipes
    [] Lack of heat
    [] Oven or stove not working
    [] Smoke detectors missing or not working
    [] Water leaks
    [] None of the above

26. How many individuals live in your household including you?
    () 1
    () 2

() 3
() 4
() 5+

27. Do you have children living in your household?
    () Yes
    () No

28. Is your household a “multi-generational household”? (Multi-generational households may include someone outside of your immediate family, such as grandparents, great-grandparents, aunts and/or uncles living with the family)
    () Yes
    () No

29. Do you take care of another person in your household who needs assistance for any reason (for example, age, illness, or disability)?
    () Yes
    () No **[SKIP TO 31]**

30. What role(s) do you play as a care partner? Please select **ALL** that apply. **[ALLOW MULTIPLE RESPONSES]**
    [] Make or attend doctor appointments
    [] Provide or arrange for transportation
    [] Provide personal care (for example help with bathing, dressing)
    [] Assist in medical decision-making
    [] Support taking medications (for example order or pick up refills, administer medicine)
    [] Other (Please specify) **[OPEN FIELD]**

31. Is English the primary language you speak at home?
    () Yes
    () No

32. Is there another language, besides English, spoken in your home?
    () Yes (Please specify) **[OPEN FIELD]**
    () No

33. In the past 12 months, has lack of reliable transportation kept you from medical appointments, meetings, work or from getting things needed for daily living?
    () Yes
    () No

34. How do you typically travel to medical appointments, meetings, work, or generally get things needed for daily living? Please select **ALL** that apply. **[ALLOW MULTIPLE RESPONSES]**
    [] I own a car and drive
    [] I rent a car and drive
    [] Someone who lives in my household drives me
    [] Someone who does not live in my household drives me

[] I use taxis or Ride Share apps (Uber, Lyft, etc.)
[] Medical transport services
[] I walk
[] I take public transport (train, bus, etc.)
[] Other **[OPEN FIELD]**

35. How far do you typically travel for a medical appointment?
    () Less than 10 minutes
    () 10-20 minutes
    () 20-30 minutes
    () More than 30 minutes
    () Most of my appointments are conducted virtually (i.e., telehealth)

36. What challenges do you have, if any, in accessing your medical care? (e.g., WiFi/bandwidth issues for telehealth) **[OPEN FIELD]**

37. In the past 12 months, has the electric, gas, oil, or water company threatened to shut off services in your home?
    () Yes
    () No
    () Already shut off

38. Within the past 12 months, you worried that your food would run out before you had enough money to buy more.
    () Often true
    () Sometimes true
    () Never true

39. How far do you typically travel for groceries?
    () Less than 10 minutes
    () 10-20 minutes
    () 20-30 minutes
    () More than 30 minutes

40. How often do you feel lonely or isolated from those around you?
    () Never
    () Rarely
    () Sometimes
    () Often
    () Always

Over the past two weeks, how often have you been bothered by any of the following problems:

41. Little interest or pleasure doing things:
    () Not at all
    () Several days
    () More than half the days
    () Nearly every day

42. Feeling down, depressed, or hopeless:
    () Not at all
    () Several days
    () More than half the days
    () Nearly every day

43. Stress means feeling tense, restless, nervous, anxious, and/or unable to sleep at night. Over the last two weeks, to what extent have you felt stressed?
    () Not at all
    () A little bit
    () Somewhat
    () Quite a bit
    () Very much

44. Review each of the following factors. On a scale of 1-4 (where is 1 is not at all and 4 is very much), tell us to what degree each of these factors causes stress [RANDOMIZE]:

Factors	1 (Not at all)	2 (A little bit)	3 (Somewhat)	4 (Very much)	Does not apply/I'm not sure
Housing instability					
Food insecurity					
Financial strain					
Transportation problems					
My health					
Health of friends/family					
Family dynamic/wellbeing					

45. Have you ever served in the U.S. Armed Forces, Reserves, or National Guard?
    () I've never served in the military
    () Only on active duty for training in the Reserves or National Guard
    () I'm now on active duty
    () Previously on active duty, but now retired
    () Prefer not to answer

Thank you very much for your time.

Supplemental Material S2. Focus group moderator's guide: key questions and overall agenda for 2-hour discussions

- **Welcome & Ground Rules (5 min)**
- **Introductions (5 min)**
- **Understanding Multiple Myeloma Disease Journey (30 min)**
    - Please briefly describe what the Multiple Myeloma disease journey has been like for you. Focus on any unique aspects of living with Multiple Myeloma, from diagnosis through treatment.
    - Discuss how, if at all, MM is unique vs. other chronic health conditions in the following areas:
        - How and where diagnosis is made
        - Number and type of care providers
        - Care setting
        - Frequency and length of appointments/stays
        - Location of appointments and required travel
        - Treatments
        - Side effects
        - Care needs/need for care partner
        - Financial issues, including cost and coverage
        - Toll on physical and emotional health
        - Disease progression and relapse
    - Quantification exercise to ask PERC members to identify areas they feel MM patients have a significantly different experience vs. other patient populations
- **Impact of Social Experiences (25 min)**
    - Introduce idea of social experiences as something that can impact health
    - Discuss impact (positive and negative) of various social experiences:
        - Health knowledge
        - Access to health information
        - Insurance coverage
        - Access to doctors & healthcare services
        - Financial stability
        - Transportation
        - Social support
        - Housing stability

    - Quantification exercise at end of discussion to ask PERC members to identify key social experiences which most (negatively) impact their care as MM patients

- **Break (5 min)**
- **Impact of Identity Experiences (25 min)**
    - Introduce idea of identity experiences as something that can impact health
    - Discuss impact (positive and negative) of various identify experiences:
        - Race or Ethnicity
        - Gender
        - Disability Status
        - Sexual Orientation
        - Age
        - Religion
        - Language
        - Culture
    - Quantification exercise at end of discussion to ask PERC members to identify key identity experiences which most (negatively) impact their care as MM patients
- **Intersectionality (20 min)**
    - Introduce idea of intersectionality
    - Have you experienced intersectionality as a part of your care journey?
    - What do you see as key ways that intersectionality impacts multiple myeloma patients?
- **Closing (5 min)**
